# Discovery of Selective Inhibitors of the *Clostridium difficile* Dehydroquinate Dehydratase

**DOI:** 10.1371/journal.pone.0089356

**Published:** 2014-02-21

**Authors:** Kiira Ratia, Samuel H. Light, Aleksandar Antanasijevic, Wayne F. Anderson, Michael Caffrey, Arnon Lavie

**Affiliations:** 1 Research Resources Center, University of Illinois at Chicago, Chicago, Illinois, United States of America; 2 Center for Structural Genomics of Infectious Diseases, Feinberg School of Medicine, Northwestern University, Chicago, Illinois, United States of America; 3 Department of Molecular Pharmacology and Biological Chemistry, Feinberg School of Medicine, Northwestern University, Chicago, Illinois, United States of America; 4 Department of Biochemistry and Molecular Genetics, University of Illinois at Chicago, Chicago, Illinois, United States of America; Institute Pasteur, France

## Abstract

A vibrant and healthy gut flora is essential for preventing the proliferation of *Clostridium difficile*, a pathogenic bacterium that causes severe gastrointestinal symptoms. In fact, most *C. difficile* infections (CDIs) occur after broad-spectrum antibiotic treatment, which, by eradicating the commensal gut bacteria, allows its spores to proliferate. Hence, a *C. difficile* specific antibiotic that spares the gut flora would be highly beneficial in treating CDI. Towards this goal, we set out to discover small molecule inhibitors of the *C. difficile* enzyme dehydroquinate dehydratase (DHQD). DHQD is the 3^rd^ of seven enzymes that compose the shikimate pathway, a metabolic pathway absent in humans, and is present in bacteria as two phylogenetically and mechanistically distinct types. Using a high-throughput screen we identified three compounds that inhibited the type I *C. difficile* DHQD but not the type II DHQD from *Bacteroides thetaiotaomicron*, a highly represented commensal gut bacterial species. Kinetic analysis revealed that the compounds inhibit the *C. difficile* enzyme with K_i_ values ranging from 10 to 20 µM. Unexpectedly, kinetic and biophysical studies demonstrate that inhibitors also exhibit selectivity between type I DHQDs, inhibiting the *C. difficile* but not the highly homologous *Salmonella enterica* DHQD. Therefore, the three identified compounds seem to be promising lead compounds for the development of *C. difficile* specific antibiotics.

## Introduction

The human pathogen *Clostridium difficile* is a spore-forming, Gram-positive, anaerobic bacillus that secretes two types of toxins, which induce severe diarrhea, fever, and nausea. Notably, the number one risk factor for *C. difficile* infection (CDI) is treatment with a broad-spectrum antibiotic to combat a preexisting bacterial infection [1]. In fact, 15–25% of all antibiotic-associated diarrhea cases are caused by *C. difficile* [2], [3]. Under normal conditions, the myriad of bacterial species in the gut successfully outcompete *C. difficile*. However, once broad-spectrum antibiotics are administered, the gut flora is largely eliminated, allowing *C. difficile* spores to proliferate without competition from the non-pathogenic bacteria [1]. In other words, the normal gut flora acts as an anti-*C. difficile* environment. Hence, physicians treating patients with CDI are challenged by two opposing goals. One goal is the eradication of *C. difficile* and the original bacterial infection for which antibiotic treatment was initiated. This would be achieved by continued administration of broad-spectrum antibiotics, with the downside of concomitant destruction of the gut flora. The other goal is cessation of antibiotic treatment to allow the recovery of the gut flora that is required to combat *C. difficile*, with the downside that ending antibiotic administration may allow *C. difficile* to proliferate in the period prior to flora recovery. This Catch-22 scenario could be resolved with a *C. difficile* specific antibiotic, which would prevent *C. difficile* proliferation while allowing for the repopulation of the gut by commensal bacteria. This concept is validated by fidaxomicin (Dificid), the first *C. difficile*-targeting narrow-spectrum antibiotic recently released to market, which has been shown to reduce the likelihood of CDI reoccurrence [4]. However, fidaxomicin affects not only *C. difficile*, being active against many Gram-positive bacteria, especially clostridia [5]. This fact, and the likely emergence of fidaxomicin-resistant *C. difficile*, indicate a clear need for additional highly *C. difficile* specific antibiotics.

Towards the goal of developing a narrow-spectrum agent for CDI, we commenced studies of shikimate pathways enzymes. The ultimate product of this 7-step pathway is chorismate, a precursor required for the biosynthesis of the three aromatic amino acids as well as other important metabolites. Because humans lack the pathway and must obtain the aromatic amino acids through dietary sources, the enzymes involved in shikimate biosynthesis provide suitable targets for antibacterial drug discovery [6]. The 3^rd^-step of the shikimate pathway involves the conversion of 3-dehydroquinate (DHQ) to 3-dehydroshikimate (DHS). Interestingly, the enzymes that catalyze this reaction, dehydroquinate dehydratases (DHQDs), are represented in bacteria by two different subtypes, I and II [7], [8]. Present in *C. difficile*, type I DHQDs are ∼30 kDa enzymes that assemble into homodimers [7] and use a Schiff-base intermediate to catalyze the dehydration reaction [9]. Present in the highly prevalent commensal bacterial species *Bacteroides thetaiotaomicron* and *Bifidobacterium longum*, among others, type II DHQDs are ∼17 kDa in size, form homododecamers, and catalyze the reaction via an enolate intermediate [7], [10]. Since type I and type II DHQDs have different binding sites and reaction mechanisms, we reasoned that it should be possible to identify small molecules that selectively inhibit the type I *C. difficile* DHQD (*cd*DHQD) but not the type II *B. thetaiotaomicron* DHQD (*bt*DHQD) and that such an inhibitor would have the desirable property of inhibiting *C. difficile* proliferation while being compatible with continued growth of a large subset of the commensal bacteria.

Here we present the discovery and characterization of three *cd*DHQD inhibitors identified by HTS. Surprisingly, in addition to exhibiting anticipated inter-type selectivity, the inhibitors also exhibit intra-type selectivity, inhibiting the *cd*DHQD but not the homologous *Salmonella enterica* type I DHQD (*se*DHQD). This work provides a proof-of-principle, demonstrating the feasibility of selectively targeting *cd*DHQD while sparing homologous enzymes from non-pathogenic bacterial species and opens the path for the development of a novel class of *C. difficile* specific antibiotics.

## Materials and Methods

### Gene Cloning and Enzyme Expression and Purification

Clostridium difficile aroD (cdDHQD), Salmonella enterica aroD (seDHQD), Vibrio cholerae aroE (V. cholerae SDH), Bacteroides thetaiotaomicron aroK (B. thetaiotaomicron SK), Bacteroides thetaiotaomicron aroQ (btDHQD), Vibrio cholerae aroQ (vcDHQD), and Yersinia pestis aroQ (ypDHQD) were amplified from genomic DNA by PCR and subcloned into the pMCSG7 expression vector. The BL21 (DE3) E. coli strain was used for recombinant expression for all but btDHQD (type II DHQD), which was expressed in the KRX E. coli strain since this enzyme was insoluble in BL21 cells. For expression, 1–3 liters of TB media were inoculated with appropriate starter culture for each protein and shaken at 225 RPM at 37°C. When an optical density of ∼0.8 at 600 nm was achieved, protein over-expression was induced by adding isopropyl-1-thio-D-galactopyranoside to a concentration of 0.5 mM, the temperature was reduced to 25°C, and the culture was left overnight. The following morning, cells were harvested by centrifugation and lysed by sonication in a buffer containing 10 mM Tris (pH 8.3), 500 mM NaCl, 10% glycerol, and 5 mM β-mercaptoethanol. The resulting lysate was cleared by centrifugation, loaded onto a 5 mL His-Trap HP Ni Sepharose column (GE Healthcare), washed with a buffer containing 10 mM Tris (pH 8.3), 500 mM NaCl, 25 mM imidazole, and 5 mM β-mercaptoethanol, and eluted in a buffer containing 10 mM Tris (pH 8.3), 500 mM NaCl, 500 mM imidazole, and 5 mM β-mercaptoethanol. The resulting elutant was injected onto a S-200 gel filtration column (GE Healthcare) equilibrated with buffer containing 10 mM Tris (pH 8.3), 500 mM NaCl, and 5 mM β -mercaptoethanol. For each purification, SDS-PAGE chromatography confirmed that the major peak off the gel filtration contained a single major band consistent in molecular weight with that anticipated for the recombinant protein. To remove expression tag, cdDHQD, seDHQD, and btDHQD were incubated overnight at 4°C with hexa-histidine tagged TEV protease, reloaded onto the 5 mL His-Trap HP Ni Sepharose column, and eluted with 25 mM imidazole.

### *Cd*DHQD High-throughput Screen

Automated high-throughput screening against a library of 50,000 Chembridge compounds was performed at room temperature on a Tecan Freedom EVO 200 liquid handling platform with an integrated Tecan Infinite F200 Pro microplate reader. All assays were performed in duplicate in black, flat-bottom 384-well plates containing a final reaction volume of 50 µL. The assays were assembled as follows: 40 µL of enzyme solution (50 mM HEPES, pH 7.5, 0.1 mg/mL BSA, 5 mM DTT, 0.01% Triton-X, 1–5 nM *cd*DHQD, 125 nM *V. cholerae* SDH, 125 nM *B. thetaiotamicron* SK) was dispensed into wells and incubated with 200 nL of 10 mM inhibitor (40 µM final concentration) for 5 min. Reactions were then initiated with 10 µL of substrate solution (50 mM HEPES, pH 7.5, 0.1 mg/mL BSA, 5 mM DTT, 0.01% Triton-X, 25 mM MgCl_2_, 5 mM ATP, 125 µM NADPH, 250 µM DHQ), shaken vigorously for 20 s, and measured for fluorescence emission intensity (excitation λ: 340 nm, emission λ: 460 nm) over a period of 7 minutes. Slopes from t = 1–5 min were recorded for each well. Each 384-well plate contained 32 positive control wells (200 nL of DMSO replacing 200 nL of compound in DMSO) and 32 negative control wells (assay components lacking *cd*DHQD). Compounds producing ≥40% inhibition were selected for further analysis.

### *Cd*DHQD Hit Validation and Selectivity Experiments

Putative hits identified in the primary screen were cherry-picked, retested in the HTS primary assay, and counter-screened against the SDH/SK coupling enzyme reaction. The counter-screen was assembled in the same manner as the primary assay, with the following changes: *cd*DHQD enzyme was eliminated from the enzyme solution, the concentration of SDH was decreased to 1 nM, and the DHQ substrate was replaced with DHS for a final concentration of 50 µM. Compounds that reproducibly inhibited *cd*DHQD and that displayed no inhibition against the coupling enzymes SK and SDH at 40 µM compound were then assayed against *se*DHQD and *bt*DHQD using the primary screen DHQD assay and up to 200 µM compound. The three confirmed hits were repurchased from Chembridge and validated against *cd*DHQD in an alternative assay monitoring the conversion of DHQ to DHS at 234 nm (50 mM potassium phosphate buffer, pH 7.5, 50 µM DHQ, 40 µM compound, 1 nM *cd*DHQD).

### Inhibitor IC_50_ and Mode of Inhibition Experiments

Inhibitor analogs were purchased from Chembridge Corporation and maintained as 50 mM stocks in DMSO. IC_50_ measurements were performed in triplicate using the primary screen assay and varying concentrations of inhibitor (0–400 µM). Reaction rates were monitored continuously for up to 30 minutes, and slopes were calculated from the linear portions of the progress curves and corrected for background signal. Data were fit to equation 1

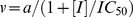
(1)using the Enzyme Kinetics module of SigmaPlot (v. 9.01 Systat Software, Inc.) where *v* is the reaction rate in the presence of inhibitor, *a* is the reaction rate in the absence of inhibitor, and [I] is the inhibitor concentration. Mode of inhibition studies with compounds **1** and **3** were performed in duplicate using the primary screen assay with varying concentrations of inhibitor and substrate. Background measurements of the assay components lacking *cd*DHQD were performed in duplicate at all substrate and inhibitor concentrations. Reaction rates were monitored continuously for up to 30 minutes, and slopes were calculated from the linear portions of the progress curves and corrected for background signal. The data were fit to the equations describing competitive, non-competitive, mixed, and uncompetitive inhibition models using the Enzyme Kinetics module of SigmaPlot.

### Fluorescence Thermal Shift (FTS) Assays

To establish dose-response relationships, compounds **1**–**3** were assayed in quadruplicate at concentrations ranging from 2.5–320 µM. Compounds **1**–**3** were added to 1.5 µg of *bt*DHQD, *cd*DHQD, or *se*DHQD and 10 nL of 5000x Sypro Orange (Invitrogen) in 10 µL of assay buffer (10 mM Tris [pH 8.4] and 150 mM NaCl). After compound addition, the plate was sealed with an optical seal, shaken, and centrifuged. The CFX384 (Bio-Rad Laboratories) real-time PCR machine was used for fluorescence detection and thermal scanning, which progressed from 10 to 95°C with a temperature ramp rate of 1.5°C/min. For analysis, the midpoint of thermal denaturation (T_m_) for each condition was calculated using an in-house software package.

### NMR Methods

NMR experiments were performed on a Bruker 900 MHz AVANCE spectrometer equipped with a cryogenic triple resonance probe. Experimental conditions were 10–15 µM DHQD, 10–500 µM compound in PBS/pH 7.2 in 90% ^1^H_2_O, 10% ^2^H_2_O (WaterLOGSY) or 100% ^2^H_2_O (STD) at 25°C. The WaterLOGSY experiments were performed as previously described [11]–[13]. Water was selectively saturated using a 2 msec square shaped pulse with a mixing time of 1–2 sec and a relaxation delay of 2.5 sec. STD experiments were performed as previously described [11], [14], [15]. In these experiments, protein ^1^H were saturated with a train of 50 msec gaussian-shaped pulses at 100 Hz power for 1 sec with “on” resonance saturation at −1 ppm and “off” resonance saturation at 30 ppm (the relaxation delay was 2.5 sec before the saturating pulses). Spectra were processed by NMRPipe with a 5 Hz line broadening function and analyzed by NMRDraw [16]. Relative %STD was defined as 100 X STD_obs_/STD_max_ where STD = ΔI/I_off_, ΔI = I_off_−I_on_ and I_off_ and I_on_ are the intensities observed for the various resonances after the “off” and “on” presaturation of HA. Errors in the WaterLOGSY and STD were estimated as ΔI/I_ref_((N_ΔI_/ΔI)^2^+(N_Ioff_/I_ref_)^2^)^0.5^ [14], where NΔI and N_Iref_ are the noise calculated by NMRDraw in the appropriate spectrum (no protein in WaterLOGSY and I_off_ in the STD). Titration data were fit to the appropriate functions using Kaleidagraph 4.1.3.

## Results

### Discovery of *cd*DHQD Inhibitors

**Figure 1:**
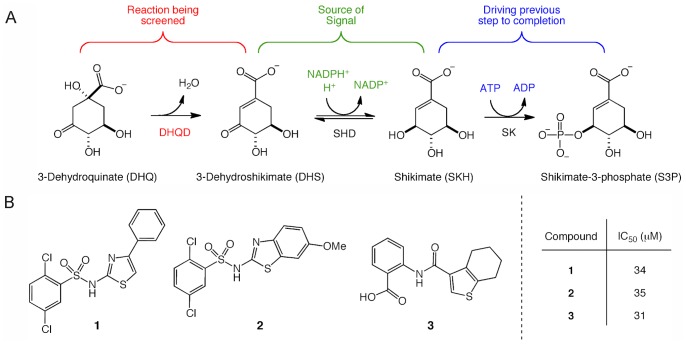
The high-throughput screen assay used to identify the three *cd*DHQD inhibitors. (A). The conversion of 3-dehydroquinate (DHQ) to 3-dehydroshikimate (DHS) by *cd*DHQD is coupled to saturating concentrations of two downstream enzymes in the shikimate pathway, shikimate dehydrogenase (SDH) and shikimate kinase (SK). The loss of NADPH fluorescence in the SDH-catalyzed step of the reaction provides a measurable, continuous readout for the assay. The presence of SK drives the reversible SDH reaction to completion and increases the dynamic range of the assay. (B) The structure of the three hit compounds and corresponding K_i_ values.

The dehydration of DHQ to DHS can be monitored spectroscopically at 234 nm due to the increased absorption of the product [17]. However, due to signal interference from library compounds at this wavelength such an assay is poorly suited for HTS. Therefore, we coupled the DHQD reaction to shikimate dehydrogenase, the subsequent step of the shikimate pathway and monitored the conversion of NADPH to NADP^+^ using a fluorescence-based assay (**Figure 1A**). During assay development we noted that the reaction was not progressing to completion and, therefore, shikimate kinase, the enzyme that follows shikimate dehydrogenase in the shikimate pathway, was added to shift the equilibrium towards the reaction product. This assay design gave an improved signal and Z’-factor of 0.68, a value indicative of a sensitive and robust assay [18].

To identify small molecule inhibitors of *cd*DHQD, a high throughput screen (HTS) of 50,000 Chembridge compounds was performed. This assay generated a low hit rate, with the vast majority of false positives resulting from compound interference with the fluorescent signal and a minority of false positives affecting the coupling enzymes. Using the direct UV assay to validate hits identified by HTS, we discovered three *cd*DHQD inhibitors (compounds **1**, **2**, and **3**) with ∼30 µM IC_50_ values (**Figure 1B**).

Hit compounds **1** and **2** exhibit striking structural similarity, both being 2,5-dichlorobenzenesulfonamide derivatives. While compound **1** incorporates a 6-substituted 2-aminobenzo[d]thiazole group, compound **2** has a 5-phenylthiazol-2-amine substituent. Interestingly, compound **3**, an amide of tetrahydrobenzo[*b*]thiophene-3-carboxylic acid, is structurally unrelated to **1** and **2**. All compounds appear to provide good starting points for future optimization in the hit-to-lead process.

### Kinetic Characterization of Hit Compounds

To better understand modes of inhibition and to determine inhibition constants (K_i_) for the three hits, we performed a complete analysis using steady state kinetics. Initial reaction rates measured at different substrate (DHQ) and compound **1** concentrations can be fit using either competitive or mixed-type inhibition models (**Figure S1A in File S1**). The K_i_ value of compound **1** to the *cd*DHQD was calculated to be 11 to 15 µM, depending on the kinetic model used. Low solubility of compound **2** prevented us from accurately determining its K_i_. Ambiguity between kinetic models was also observed for compound **3,** for which K_i_ values ranged between 19 and 22 µM depending of the kinetic model used (**Figure S1B in File S1**).

In the competitive model, the inhibitor (I) binds to the enzyme (E) but not the enzyme-substrate (ES) complex – only EI is formed. In contrast, in the mixed inhibition model, the inhibitor binds to both E and ES complex, forming EI and ESI. Competitive inhibition is characterized by an increased apparent K_m_ but unchanged V_max_, as increased substrate concentration will shift EI to ES, negating the inhibition. For mixed inhibition, while the effect due to the presence of the inhibitor on apparent K_m_ can vary, V_max_ is decreased – even at high [S], the presence of ESI remains, reducing V_max_. Thus, this differential effect on V_max_ can be used to distinguish between competitive and mixed models of inhibition. To clarify the mechanism of inhibition, we re-assayed *cd*DHQD with substrate concentrations reaching as high as 5 mM, asking if the V_max_ differs to that observed in the absence of inhibitor. Indeed, in the presence of 200 µM of compound **3** the apparent K_m_ increased 10-fold but V_max_ was unchanged (results not shown). This result is consistent with a competitive mechanism of inhibition and is counter to the mixed inhibition model.

### Selectivity of Compounds 1–3

To determine inhibitor selectivity, we screened compounds **1**–**3** against a panel of purified DHQDs that included the type II *bt*DHQD, type II *vc*DHQD, type II *yp*DHQD, and type I *se*DHQD. *Se*DHQD shares 56% sequence identity (78% sequence homology) and a highly homologous quaternary structure (backbone root mean square deviation of homodimer = 1.2 Å) with *cd*DHQD [19]. As anticipated, the kinetic assays revealed that the compounds were inactive against the structurally and mechanistically distinct type II DHQDs, but, surprisingly, also inactive against the related type I *se*DHQD (**Table 1**).

**Table 1:** IC_50_s for Multiple DHQDs (µM).

	Compound 1	Compound 2	Compound 3
**Type I**			
*cd*DHQD	34	35	31
*se*DHQD	>200	>200	>200
**Type II**			
*bt*DHQD	>200	>200	>200
*vc*DHQD	>200	>200	>200
*yp*DHQD	>200	>200	>200

**Figure 2:**
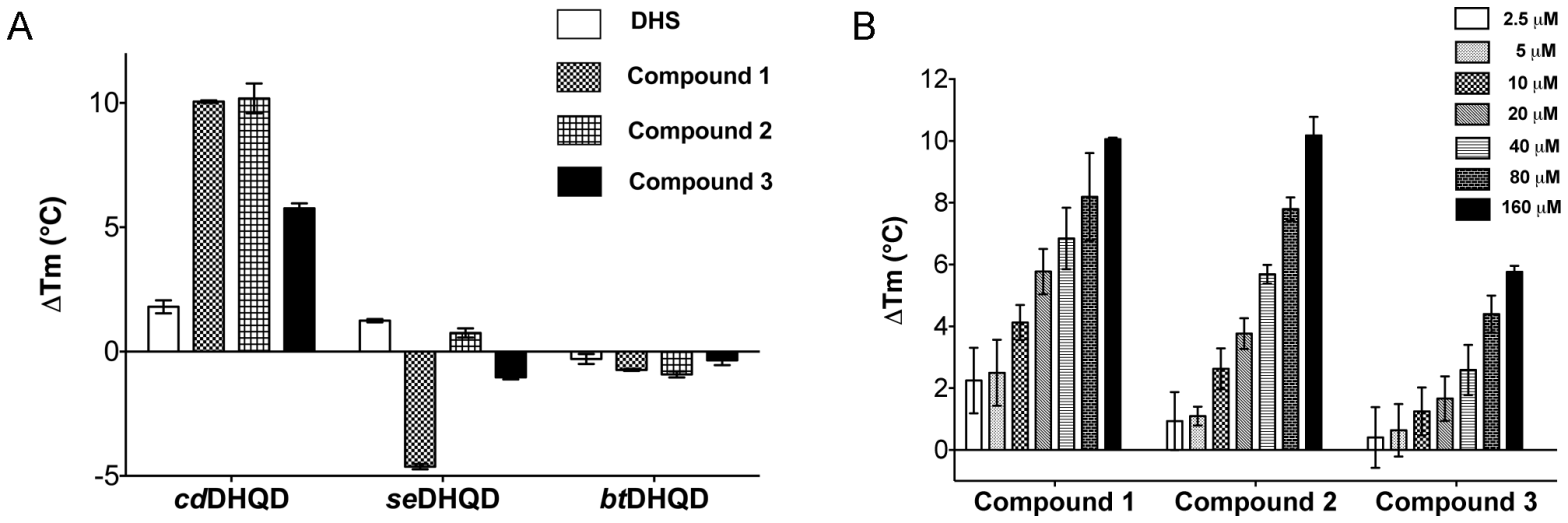
Fluorescence Thermal Shift demonstrates that Compounds 1–3 bind selectively to *cd*DHQD. (A) The effect of 160 µM DHS/compounds **1**–**3** on the thermal stability of *cd*DHQD, *se*DHQD, and *bt*DHQD as determined by FTS. The ΔT_m_ represents the difference in midpoint thermal denaturation temperature compared to the ligand-less control. (B) FTS experiments show ΔT_m_ of *cd*DHQD as a function of compound **1**–**3** concentration.

**Figure 3:**
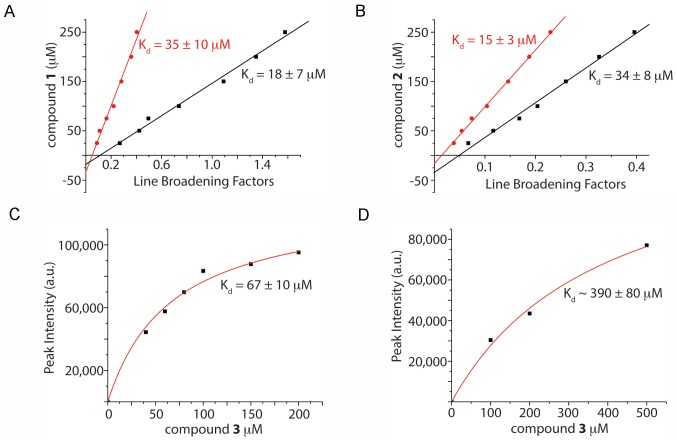
NMR demonstrates that Compounds 1–3 bind selectively to *cd*DHQD. NMR titration of (A) compound **1,** (B) compound **2**, (C) compound **3** to *cd*DHQD. All three compounds demonstrate binding to *cd*DHQD with K_d_ values of ∼25 µM for compounds **1** & **2**, and ∼65 µM for compound **3**. (D) NMR titration of compound **3** to *se*DHQD reveals much reduced affinity, with a K_d_ of ∼400 µM binding. In (A) and (B), the red and black lines represent the results from two different resonances.

Next, we turned to two biophysical techniques to confirm the unexpected intra-type selectivity. *First*, Fluorescence Thermal Shift (FTS) assays were performed to probe the effect of compounds **1**–**3** on *cd*DHQD, *bt*DHQD, and *se*DHQD thermal stability. Consistent with selective *cd*DHQD binding, all three compounds thermally stabilized *cd*DHQD, but not *bt*DHQD and *se*DHQD, in a dose-dependent manner (**Figure 2**). *Second*, NMR spectroscopy was used to measure compounds **1**–**3** resonances in the presence and absence of enzyme. In the case of compounds **1** and **2**, we observed line broadening of the compound resonances upon addition of *cd*DHQD, which is characteristic of chemical exchange in the intermediate time scale (**Figure S2A in File S1**). The double reciprocal plot of the line broadening effects yields a K_d_ of ∼27 µM for binding of compound **1** and K_d_ of ∼25 µM for binding of compound **2** to *cd*DHQD (**Figure 3A & 3B**). Importantly, little line broadening was observed for *se*DHQD at similar compound concentrations (<1 Hz vs. >6 Hz for *cd*DHQD, **Figure S2B**), preventing the calculation of a K_d_ and suggesting that compounds **1** and **2** bind with significantly lower affinity to *se*DHQD.

Due to a faster exchange rate, the resonances of compound **3** were not measurably broadened upon the addition of *cd*DHQD. Accordingly, we chose the WaterLOGSY NMR experiment, which has previously been shown to be useful for small molecule binding to proteins in fast exchange [11]–[13], to assay binding of compound **3** to DHQD. The dependence of the WaterLOGSY signal on concentration allowed us to determine a K_d_ of ∼65 µM for binding of compound **3** to *cd*DHQD (**Figure 3C**). Importantly, in the case of *se*DHQD, the affinity for compound **3** is again significantly lower (**Figure 3D** **and Figure S3 in File S1**), confirming its selectivity for *cd*DHQD.

### NMR Competition Experiments Suggest a Shared Binding Site for Compounds 1–3

Due to the structural similarity between compounds **1–3**, we next tested whether they bind to overlapping sites on *cd*DHQD using an NMR-based competition assay. This experiment probes how inhibitors affect product binding to the enzyme. We first incubated the enzyme with substrate allowing the reaction to reach equilibrium – the reaction is monitored by comparing the relative intensity of substrate and product resonances. In this way we determined that at equilibrium, the ratio between substrate/product to be ∼1∶20, a result consistent with literature reports that also found a high equilibrium ratio [20]. Since the substrate to product ratio is skewed towards the products at equilibrium conditions we decided to evaluate the effect of inhibitors on product resonances.

**Figure 4:**
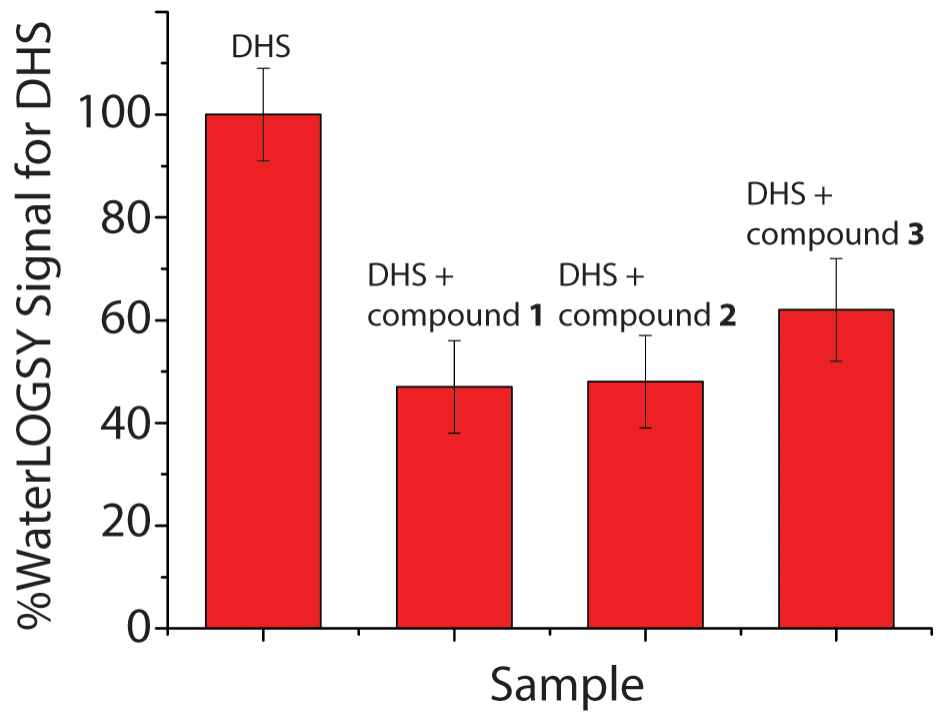
NMR competition experiments to characterize the binding site of compounds 1–3 on *cd*DHQD. WaterLOGSY NMR competition experiment between compounds **1–3** and product DHS in the presence of *cd*DHQD. The reduced WaterLOGSY signal for DHS in the presence of the compounds indicate that the compounds compete with DHS for binding to *cd*DHQD.

Hence, prior to addition of the compounds, we incubated the enzyme in the presence of substrate until equilibrium was established, and subsequently monitored the resonances of the product DHS. As shown in **Figure 4**, the addition of compounds **1–3** reduces DHS binding by 40–50%. This result is consistent with all three compounds binding to the product binding site on *cd*DHQD (primary data are presented in **Figure S4 in File S1**). However, this experiment by itself does not exclude the possibility of the inhibitors binding to an allosteric binding site, potentially even different allosteric sites for each compound.

### NMR Experiments to Map the Compound-DHQD Interaction

**Figure 5:**
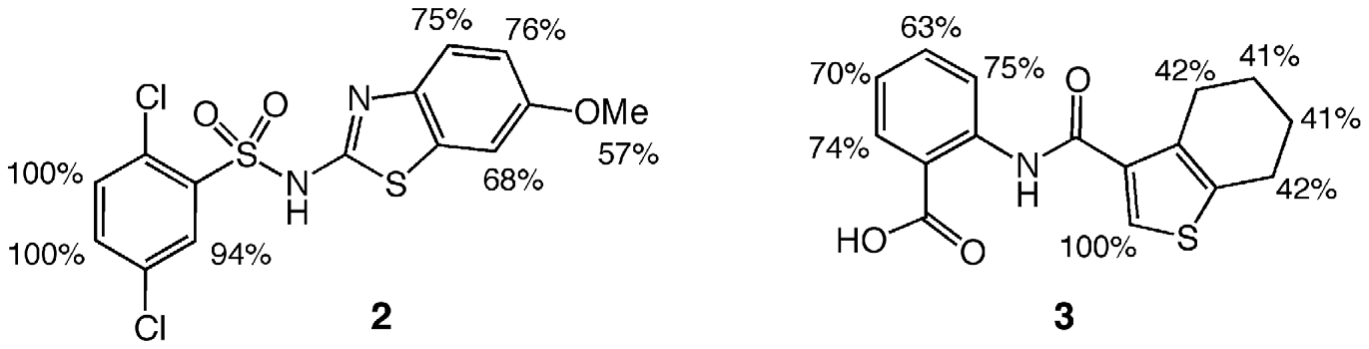
STD NMR experiments map the binding of compounds 2 and 3 on *cd*DHQD. The relative proximity of compounds’ carbons to *cd*DHQD atoms based on STD NMR data is shown.

Saturation Transfer Difference (STD) NMR has been shown to be useful for identifying the ligand ^1^H in closest proximity to the protein surface [11], [14], [15]. Accordingly, we used the technique to characterize the interactions of compounds **2** and **3** with *cd*DHQD (compound **1** was not amenable to this assay due to line-broadening and weak signal). A summary of these experiments is shown in **Figure 5**. In the case of compound **2**, the relative STD signals range from 57–100%, with protons from the dichlorobenzenesulfonamide substituent producing reliably stronger signals then those from the phenylthiazol core. In the case of compound **3**, the relative STD signals range from 41–100%, with the thiophene proton producing the strongest signal, followed by protons on the phenyl ring. Interestingly, protons on compound **2**’s dichlorobenzenesulfonamide substituent and compound **3**’s phenyl group, as well as compound **2**’s phenylthiazol and compound **3**’s tetrahydrobenzo[*b*]thiophene group, exhibit comparable relative intensities, raising the possibility that the two compounds similarly engage *cd*DHQD.

### Structure-activity Analysis

To identify the specific elements required for DHQD inhibition, we tested analogs of compounds **1**–**3** (**Figure S5A in File S1**). For compounds **1** and **2**, modifications to the dichlorobenzenesulfonamide core were poorly tolerated. Analogs lacking the two chlorines were completely inactive and replacing the chlorines with fluorines resulted in much reduced inhibition. Adding a third chlorine at the ortho position also abolished inhibition suggests that the dichlorobenzenesulfonamide core makes critical and easily disrupted interactions with the enzyme. As is evident from the differences between compounds **1** and **2**, modifications to the thiazole substituent were better tolerated. While a compound containing the unmodified thiazole ring was inactive, a variety of compounds with distinct additions to the thiazole substituent displayed similar activity to the preliminary hits (**Figure S5B in File S1**).

## Discussion

To our knowledge, the compounds described here represent the first non-mechanism based type I DHQD inhibitors identified to date. Moreover they exhibit properties of inter- and intra-type specificity desirable of a narrow-spectrum antibiotic for CDI treatment. While the identification of selective *cd*DHQD inhibitors provides a proof-of-concept, additional studies are required to optimize inhibitor binding. In principle, the present set of studies provides insight that should be useful for the development of high affinity inhibitors. In particular, the STD-NMR data suggests that compound **1** and **2**’s dichlorophenyl core intimately associates with *cd*DHQD and SAR deduced from the analysis of inhibitor analogs confirm its importance for binding. By contrast, these approaches suggest that modified thiazole substituent is less intimately associated with the protein and less critical for binding. These observations are consistent with the existence of a region adjacent to the thiazole binding site that can be viably expanded into – arguably making substitutions at the thiazole ring to be the most promising strategy for inhibitor optimization.

It is clear the structural information about inhibitor binding could help explain the observed specificity and further aid the development of high affinity inhibitors. Despite attempting multiple co-crystallization and soaking experiments, we were unable to obtain inhibitor bound crystal complexes. In the absence of a complex crystal structure, inferences about the basis of inhibitor binding can be made from available structural and kinetic data. Previously described crystal structures reveal DHQD adopts two discrete conformational states [21]. In the absence of ligand the functionally important β8-α8 loop adopts an open and partially disordered conformation. In liganded structures, closure of the β8-α8 loop establishes hydrogen bonding interactions with the lysine171 Schiff base-bound reaction intermediate [19].

Three observations suggest that the inhibitors, unlike the reaction intermediate, bind DHQD’s open loop conformational state: First, whereas co-crystallization and crystal soaking experiments readily yielded reaction intermediate bound complexes, to date, we have been unable to obtain inhibitor bound complexes, as noted above (data not shown). If the substrate and inhibitor bind the same conformational state, then successful soaks with the comparable affinity inhibitors could reasonably be expected. Second, with loop closure the reaction intermediate is buried beneath the protein surface [21]. As the active site snuggly fits the reaction intermediate, it is difficult to envision how it could accommodate the appreciably larger structures of compounds **1–3**. Finally, *cd*DHQD and *se*DHQD active sites are virtually structurally identical in the closed β8-α8 loop conformational state. Such structurally similar binding sites are inconsistent with the drastically differing binding affinities the compounds exhibit for the two enzymes.

Collectively, these observations make a strong case that compounds **1–3** do not bind the closed loop conformational state. The size of the compounds necessitates that they bind the open loop conformational state and extend out of the active site. This fact could account for the specificity of the compounds to *cd*DHQD. A number of residues on the perimeter of the active site differ between *cd*DHQD and *se*DHQD and interactions with one or more of these likely accounts for the observed intra-type specificity. Future work will focus on testing the ability of these compounds to inhibit the growth of *C. difficile*, while at the same time, sparring a large subset of the commensal flora.

In sum, this work demonstrates that selective inhibition of *cd*DHQD is possible, and that the hits identified bind at the active site, and in so doing, likely prevent the β8-α8 loop from closing. The interactions made with residues more distant to the active site are likely responsible for the selectivity towards the *C. difficile* enzyme versus the highly homologous *S. enterica* enzyme. We are currently synthesizing analogs of compounds **1**–**3** in order to identify molecules that bind with higher affinity, but that also maintain a preference for *cd*DHQD. Such compounds would hold promise for CDI therapy.

## Supporting Information

File S1 **This file contains Figure S1–Figure S5.** Figure S1, Fit of initial rate measured under varying substrate and inhibitor concentrations to the mixed-type and competitive models. (A) Fits for compound 1. (B) Fits for compound 3. Figure S2, NMR data for binding of compounds 1 and 2 to *cd*DHQD. (A) Line broadening observed for compound 1 upon addition of *cd*DHQD or *se*DHQD. (B) Line broadening observed for compound 2 upon addition of *cd*DHQD or *se*DHQD. Note that line broadening was observed only in the presence of *cd*DHQD but not with *se*DHQD, demonstrating the selectivity of the compounds to *cd*DHQD. The experimental conditions were 10 µM DHQD, 100 µM compound in PBS/pH 7.2, 10% ^2^H_2_O at 25°C. Data were acquired on a Bruker 900 MHz spectrometer equipped with a cryogenic triple resonance probe. Line widths were estimated using NMRDraw (Delaglio et al., 1995). Figure S3, NMR data for binding of compound 3 to *cd*DHQD. The experimental conditions were 10 µM DHQD, 100 µM compound 3 in PBS/pH 7.2, 10% ^2^H_2_O at 25°C. Data were acquired on a Bruker 900 MHz spectrometer equipped with a cryogenic triple resonance probe. The WaterLOGSY experiments employed a 1 sec mixing time. Figure S4, NMR data for the WaterLOGSY competition assay. Experimental conditions were 10 µM DHQD, 100 µM DHS and 100 µM compounds 1, 2 or 3 in PBS/pH 7.2, 10% ^2^H_2_O at 25°C. Data were acquired on a Bruker 900 MHz spectrometer equipped with a cryogenic triple resonance probe with a WaterLOGSY mixing time of 2 sec. Figure S5, Structure-activity relationship analysis for compounds 1–3. (A), (B), and (C): Inhibition of the *cd*DHQD catalyzed dehydration reaction was measured for compounds 1–3 and select analogs. The critical role for binding through the phenyl aryl chlorine atoms (compounds 1 & 2) and the carboxamideylic moiety (compound 3) is apparent. (D) A series of analogs demonstrate that distinct additions to the dichlorobenzenesulfonamide thiazole core shared by compounds 1 and 2 have little effect on IC_50_.
(DOCX)
