# Supplementary material for: Discovery of Selective Inhibitors of the Clostridium difficile Dehydroquinate Dehydratase
Source: PLoS One. 2014 Feb 21;9(2):e89356. doi: 10.1371/journal.pone.0089356 (PMC3931744; doi:10.1371/journal.pone.0089356)
Supplement: File S1 — This file contains Figure S1–Figure S5. Figure S1, Fit of initial rate measured under varying substrate and inhibitor concentrations to the mixed-type and competitive models. (A) Fits for compound 1. (B) Fits for compound 3. Figure S2, NMR data for binding of compounds 1 and 2 to cdDHQD. (A) Line broadening observed for compound 1 upon addition of cdDHQD or seDHQD. (B) Line broadening observed for compound 2 upon addition of cdDHQD or seDHQD. Note that line broadening was observed only in the presence of cdDHQD but not with seDHQD, demonstrating the selectivity of the compounds to cdDHQD. The experimental conditions were 10 µM DHQD, 100 µM compound in PBS/pH 7.2, 10% 2H2O at 25°C. Data were acquired on a Bruker 900 MHz spectrometer equipped with a cryogenic triple resonance probe. Line widths were estimated using NMRDraw (Delaglio et al., 1995). Figure S3, NMR data for binding of compound 3 to cdDHQD. The experimental conditions were 10 µM DHQD, 100 µM compound 3 in PBS/pH 7.2, 10% 2H2O at 25°C. Data were acquired on a Bruker 900 MHz spectrometer equipped with a cryogenic triple resonance probe. The WaterLOGSY experiments employed a 1 sec mixing time. Figure S4, NMR data for the WaterLOGSY competition assay. Experimental conditions were 10 µM DHQD, 100 µM DHS and 100 µM compounds 1, 2 or 3 in PBS/pH 7.2, 10% 2H2O at 25°C. Data were acquired on a Bruker 900 MHz spectrometer equipped with a cryogenic triple resonance probe with a WaterLOGSY mixing time of 2 sec. Figure S5, Structure-activity relationship analysis for compounds 1–3. (A), (B), and (C): Inhibition of the cdDHQD catalyzed dehydration reaction was measured for compounds 1–3 and select analogs. The critical role for binding through the phenyl aryl chlorine atoms (compounds 1 & 2) and the carboxamideylic moiety (compound 3) is apparent. (D) A series of analogs demonstrate that distinct additions to the dichlorobenzenesulfonamide thiazole core shared by compounds 1 and 2 have little effect on IC50. (DOCX) [file pone.0089356.s001.docx]

**Supplementary Material**

**Discovery of selective inhibitors of the *Clostridium difficile* dehydroquinate dehydratase**

Kiira Ratia^a,*^, Samuel H. Light^b,c,*^, Alexander Antansijevic^d,*^, Wayne F. Anderson^b,c^, Michael Caffrey^d^, and Arnon Lavie^d^

^a^Research Resources Center, University of Illinois at Chicago, Chicago, Illinois, USA

^b^Center for Structural Genomics of Infectious Diseases, Feinberg School of Medicine, Northwestern University, Chicago, Illinois, USA

^c^Department of Molecular Pharmacology and Biological Chemistry, Feinberg School of Medicine, Northwestern University, Chicago, Illinois, USA

^d^Department of Biochemistry and Molecular Genetics, University of Illinois at Chicago, Chicago, Illinois, USA.

*authors contributed equally to this work

| A | 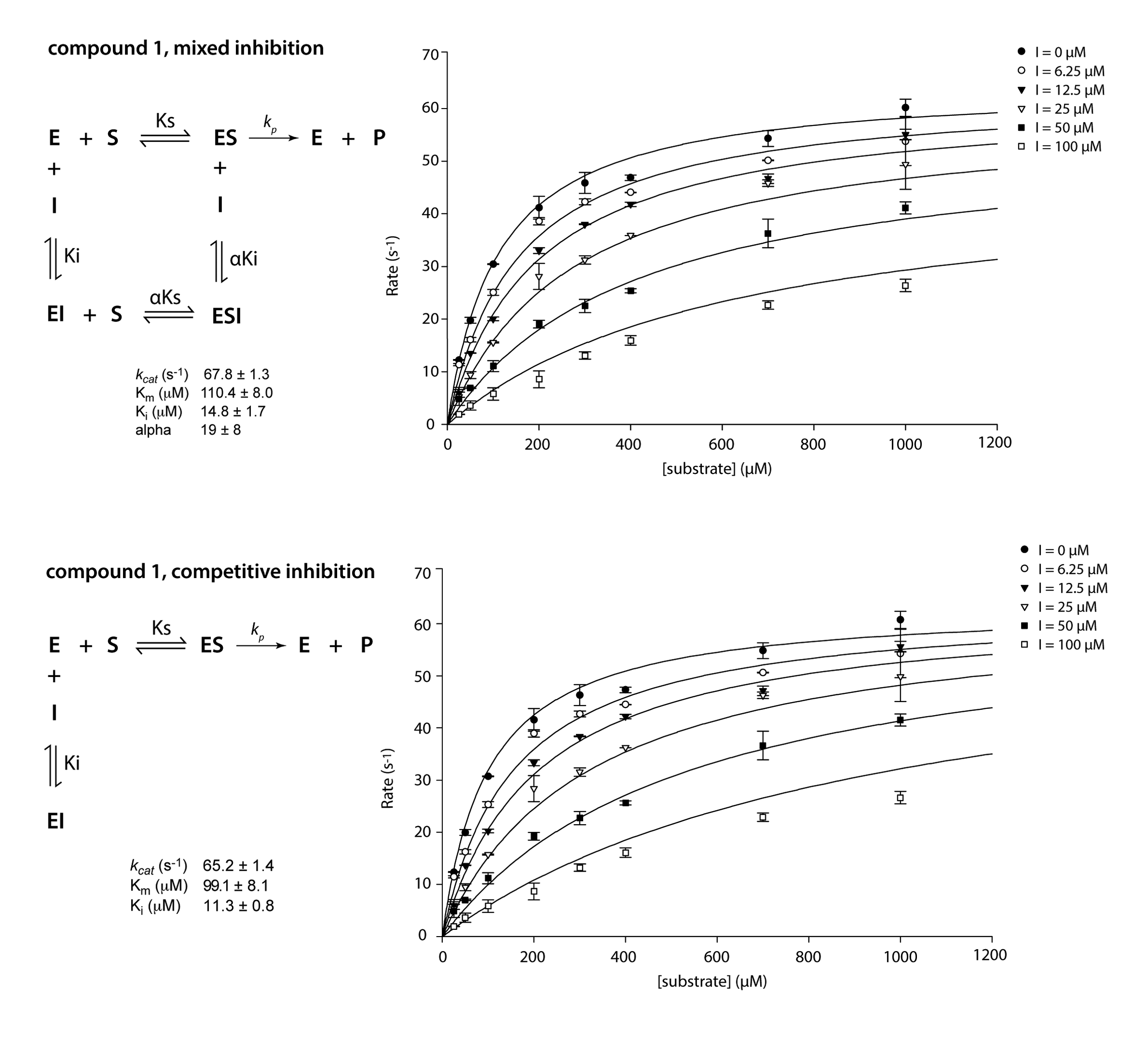 |
| --- | --- |
| B | 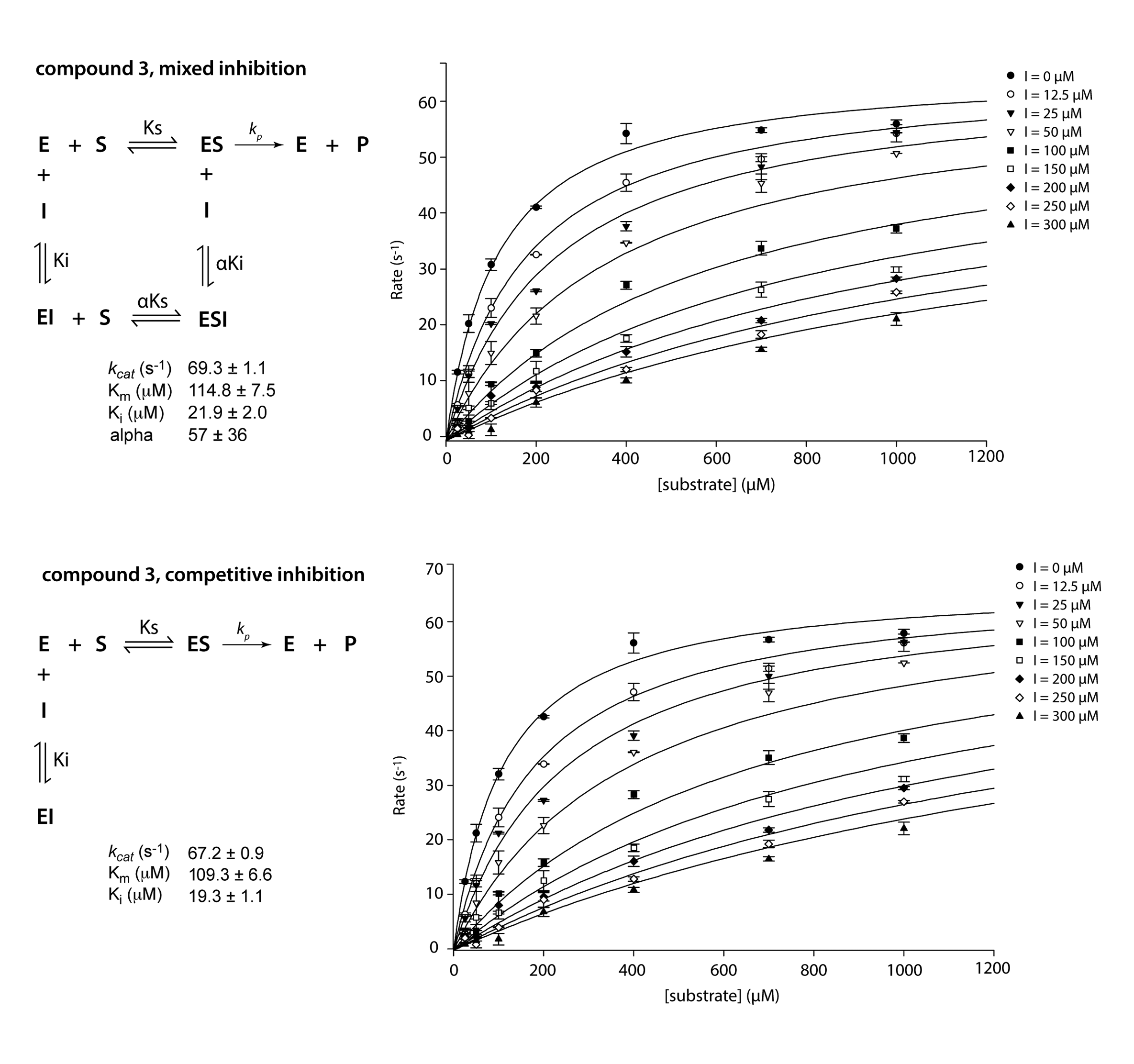 |

**Supplementary Figure S1. Fit of initial rate measured under varying substrate and inhibitor concentrations to the mixed-type and competitive models.** (A) Fits for compound **1**. (B) Fits for compound **3**.

| A | 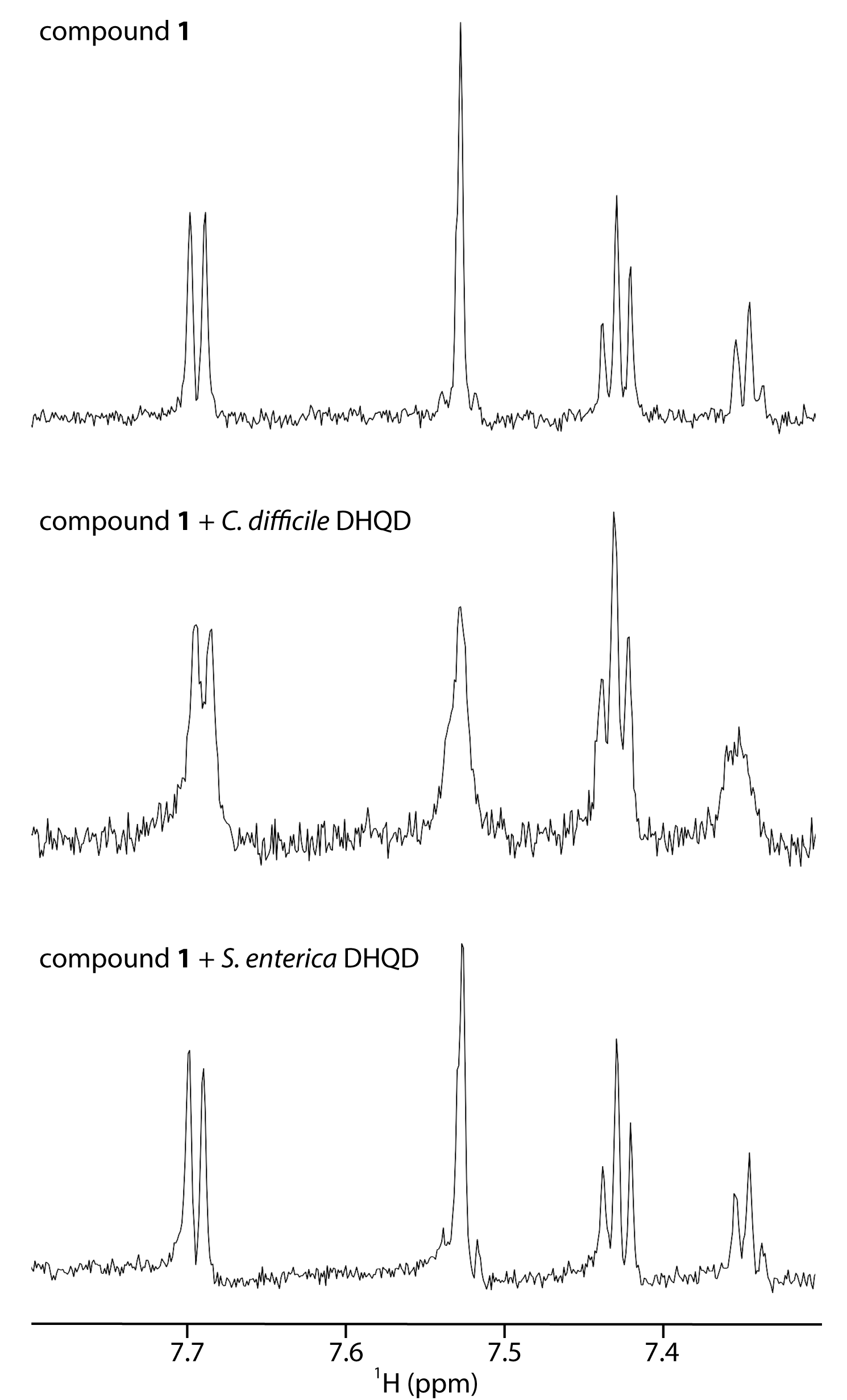 | B | 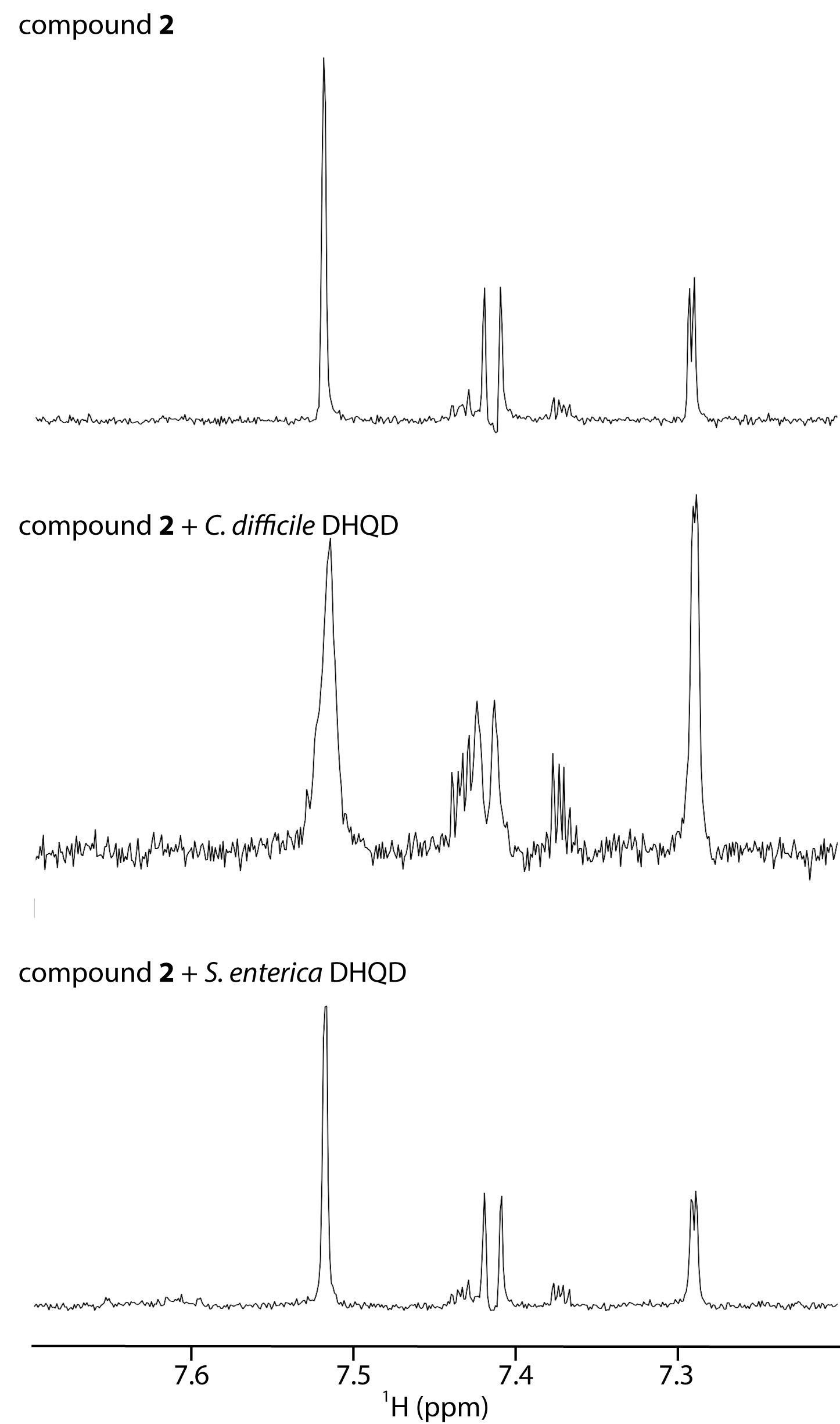 |
| --- | --- | --- | --- |

**Supplementary Figure S2. NMR data for binding of compounds 1 and 2 to *cd*DHQD.** (A) Line broadening observed for compound **1** upon addition of *cd*DHQD or *se*DHQD. (B) Line broadening observed for compound **2** upon addition of *cd*DHQD or *se*DHQD. Note that line broadening was observed only in the presence of *cd*DHQD but not with *se*DHQD, demonstrating the selectivity of the compounds to *cd*DHQD. The experimental conditions were 10 μM DHQD, 100 μM compound in PBS/pH 7.2, 10% ^2^H_2_O at 25˚C. Data were acquired on a Bruker 900 MHz spectrometer equipped with a cryogenic triple resonance probe. Line widths were estimated using NMRDraw (Delaglio et al., 1995).


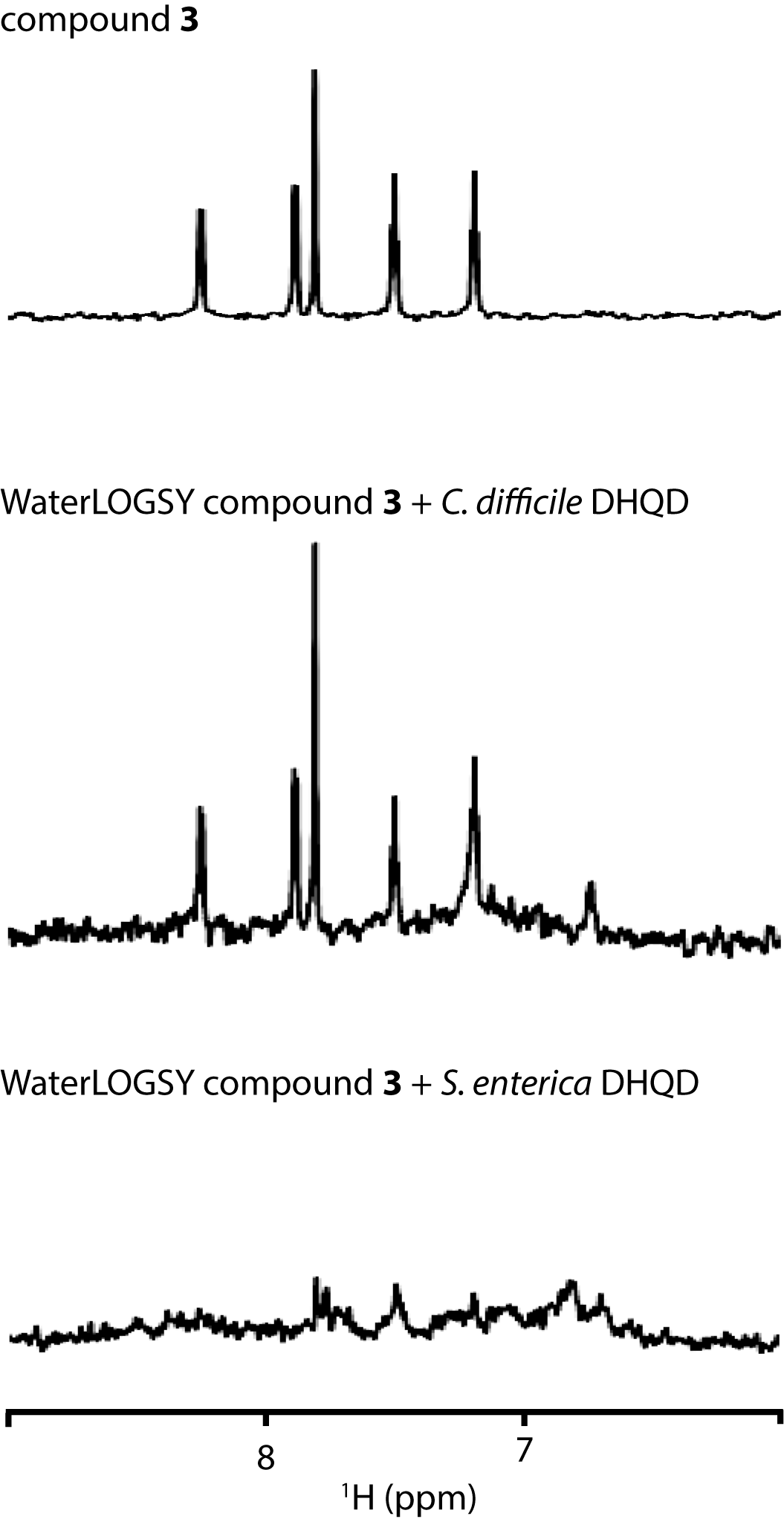


**Supplementary Figure S3. NMR data for binding of compound 3 to *cd*DHQD.** The experimental conditions were 10 μM DHQD, 100 μM compound **3** in PBS/pH 7.2, 10% ^2^H_2_O at 25˚C. Data were acquired on a Bruker 900 MHz spectrometer equipped with a cryogenic triple resonance probe. The WaterLOGSY experiments employed a 1 sec mixing time.


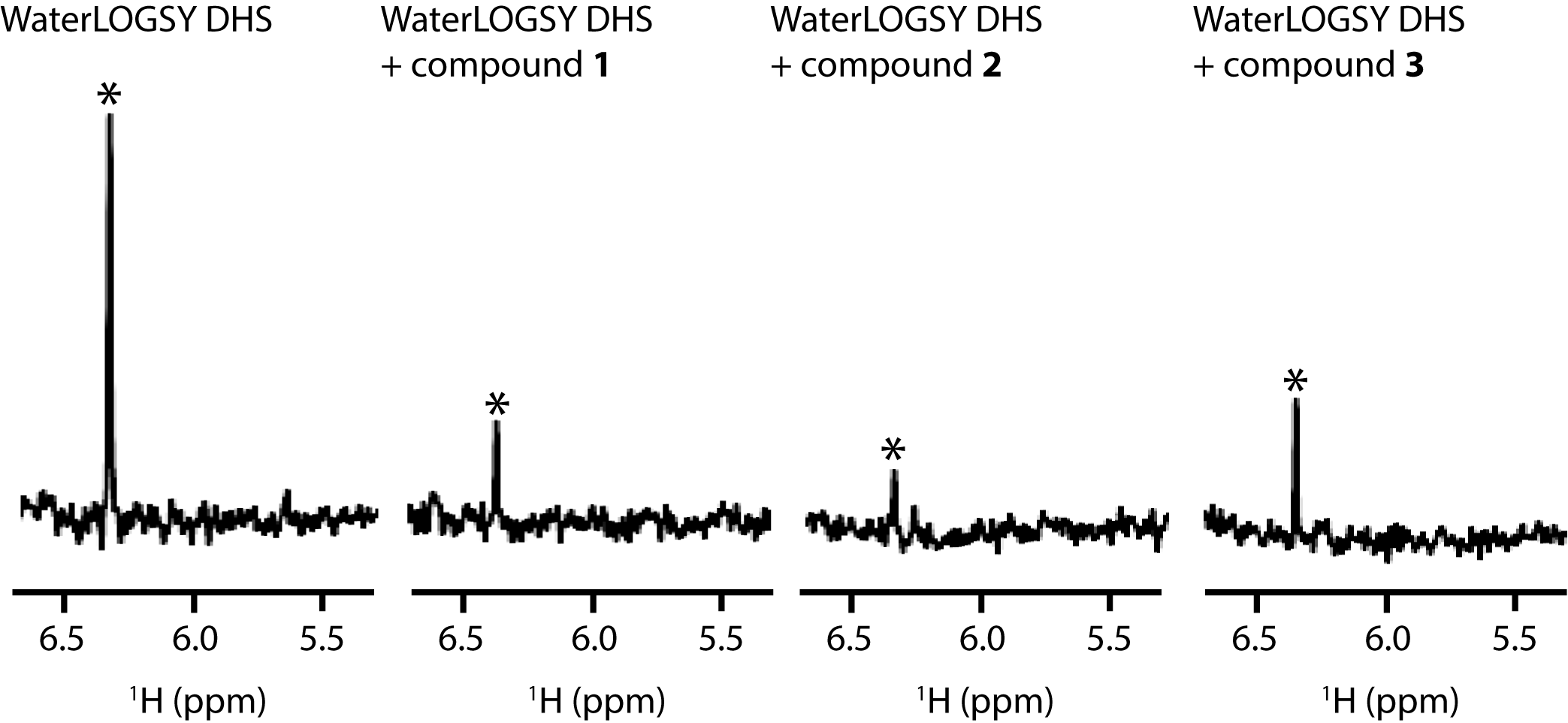


**Supplementary Figure S4: NMR data for the WaterLOGSY competition assay.** Experimental conditions were 10 μM DHQD, 100 μM DHS and 100 μM compounds **1**, **2** or **3** in PBS/pH 7.2, 10% ^2^H_2_O at 25˚C. Data were acquired on a Bruker 900 MHz spectrometer equipped with a cryogenic triple resonance probe with a WaterLOGSY mixing time of 2 sec.





**D**





**Supplementary Figure S5. Structure-activity relationship analysis for compounds 1-3.** (A), (B), and (C): Inhibition of the *cd*DHQD catalyzed dehydration reaction was measured for compounds **1-3** and select analogs. The critical role for binding through the phenyl aryl chlorine atoms (compounds **1** & **2**) and the carboxamideylic moiety (compound **3**) is apparent. (D) A series of analogs demonstrate that distinct additions to the dichlorobenzenesulfonamide thiazole core shared by compounds **1** and **2** have little effect on IC_50_.
